# Editorial: Current challenges in photosynthesis: From natural to artificial, volume II

**DOI:** 10.3389/fpls.2022.1113693

**Published:** 2023-01-05

**Authors:** Harvey J. M. Hou, Mohammad M. Najafpour, Suleyman I. Allakhverdiev, Govindjee Govindjee

**Affiliations:** ^1^ Laboratory of Forensic Analysis and Photosynthesis, Department of Physical and Forensic Sciences, Alabama State University, Montgomery, AL, United States; ^2^ Department of Chemistry, Center of Climate Change and Global Warming, Institute for Advanced Studies in Basic Sciences, Zanjan, Iran; ^3^ Controlled Photobiosynthesis Laboratory, Institute of Plant Physiology, Russian Academy of Sciences, Moscow, Russia; ^4^ Department of Biochemistry, and Center of Biophysics and Quantitative Biology, University of Illinois at Urbana-Champaign, Urbana, IL, United States; ^5^ Department of Plant Biology, and Center of Biophysics and Quantitative Biology, University of Illinois at Urbana-Champaign, Urbana, IL, United States

**Keywords:** photosynthesis, artificial photosynthesis, structure, function, photosystem, light harvesting

Photosynthesis, as one of the most important chemical reactions, has powered our planet for over four billion years on a massive scale, in particular *via* water-splitting reactions (Shevela et al., 2019; Blankenship, 2021). It captures and stores solar energy through a remarkable oxygenic photosynthetic machinery in plants, algae, and cyanobacteria, as well as through an anoxygenic machinery in photosynthetic bacteria. Photosynthesis research is a truly interdisciplinary field involving physics, chemistry, biology, engineering, and computer science. The molecular mechanisms of the fascinating photosynthetic machine have been extensively investigated using a combination of spectroscopy, mutagenesis, and structural approaches (Bryant, 1994; Blankenship et al., 1995; Ort and Yocum, 1996; Green and Parson, 2003; Wydrzynski and Satoh, 2006; Golbeck, 2010; Hou et al., 2017; Shen et al., 2021). To address global energy and climate issues, tremendous efforts have been made using the principles of natural photosynthesis *via* artificial photosynthesis, and bioenergy applications worldwide (Barber, 2009; Bruno, 2016; Najafpour et al., 2016; Brudvig and Campagna, 2017; Hou et al., 2017; Blankenship, 2021).

In 2014, a group of international scientists attempted to target the challenges in natural and artificial photosynthesis (Hou et al., 2014). There, we presented advances in both natural and artificial photosynthesis with 10 papers authored by 31 scientists from Australia, Finland, Germany, Sweden, Taiwan, and the USA. This publication provided the readers with exciting new results, their implications, as well as their potential limitations; further, it addressed new open questions.

Since 2014, new molecular details of natural photosynthesis and outstanding applications *via* artificial photosynthesis have become available. For example, strategies to reduce the antenna size were developed to improve photosynthetic efficiency (Ort et al., 2015; Negi et al., 2020; Blankenship, 2021). In addition, synthetic chemical approaches were utilized in semiconductor-based light-driven electron transfer systems, as well as in artificial water-splitting systems (Brudvig and Campagna, 2017; Hou et al., 2017; Zhang and Sun, 2019).

Invited and encouraged by the Frontiers Editorial Team, the current Research Topic, “Challenges in photosynthesis: From natural to artificial,” Volume II, was organized with the goal to provide an update on natural and artificial photosynthesis from 2014 through 2022. Due to the extremely tight time frame and other unexpected reasons, many of the contributors were unable to submit their manuscripts at this time. We intend to provide a more comprehensive version of the current Topic, as Volume III, in the future.

Although the current update (in Volume II) is relatively short, yet it has three thorough reviews and two original research papers. It is noteworthy that these five papers are written by 34 authors from seven countries: China (5), France (2), Israel (3), Netherlands (5), Switzerland (4), Thailand (13), and Taiwan (2).

In natural photosynthesis, several photosynthetic membrane proteins play vital roles in regulating solar energy conversion. For example, cytochrome (Cyt) *b*-559 is a crucial component of the Photosystem II complex for its appropriate functioning and assembly. In the opening review article, Chiu and Chu present a timely review of the functional roles of cytochrome *b*-559 in Photosystem II (PS II); this review includes a discussion of new exciting results on site-directed mutagenesis and high-resolution structures of native, inactive, and assembly intermediates of PS II complexes. Further, the novel results presented in this review offer an in-depth understanding of the structure and the mechanisms of Cyt *b*-559 in PS II.

Synthetic mimics of the photosynthetic oxygen evolution is a fast-growing area in artificial photosynthesis. Recent advances have been achieved using X-ray crystallography (Shen et al., 2021). In the second review paper, Chen et al. evaluate the synthetic Mn_4_XO_4_ clusters (X=Ca/Y/Gd) to mimic the geometric structure, the electronic structures, and the redox property of PSII oxygen-evolving complex. These results are extremely interesting and are expected to provide the structural molecular platform to investigate the molecular details of the photosynthetic water oxidation chemistry.

In the third review article, Shlosberg et al. summarize the application of photosynthesis in the area of bioenergy, using cyanobacteria, green algae, seaweeds, and plants, to produce electricity. Here, we have the description of bio-photoelectrochemical cells, achieved through the combination of native photosynthesis with electrodes and electron mediators. This exciting ‘*green energy’* approach can be used in various systems, including cyanobacteria, green algae, seaweeds, and higher plants, for light harvesting and energy production. Furthermore, in this review, future challenges of bio-photoelectrochemical cells, for practice applications, are presented.

In the first original research paper in this Volume, Vayghan et al. report that the stable loss of the LHCB1, a component of the light-harvesting complex II (LHCII) complex, induces compensatory mechanisms in *Arabidopsis thaliana;* these authors used CRISPR/Cas9 to knock out five genes (*Lhcb1.1, Lhcb1.2, Lhcb1.3, Lhcb1.4*, and *Lhcb1.5*) encoding LHCB1 and found that the loss of LHCB1 drastically altered the thylakoid structure. The method presented in the paper has the potential to be used to improve photosynthetic efficiency for crop productivity.

In the last paper in this Volume, Chutimanukul et al. examined the physiological responses in secondary metabolite production in the herbal plant, the holy basil *Ocimum tenuiflorum* L, under controlled environmental conditions. The methods described in the paper are suggested to produce high-quality raw materials using this herbal plant as a plant factory for the food and pharmaceutical industries.

The volume II of the Research Topic provides some of the most recent updates in the field of photosynthesis from natural to artificial, including the aspects of methodology, structure, mechanism, and applications. We hope the readers may benefit from the work presented here to stimulate their research innovation, promote new discoveries and breakthroughs to understand the amazing working details of photosynthesis, and offer effective strategies to address the issues of food, energy, and climate change worldwide.

## Author contributions

All authors listed have made a substantial, direct, and intellectual contribution to the work and approved it for publication.
